# Conjugation with Dihydrolipoic Acid Imparts Caffeic Acid Ester Potent Inhibitory Effect on Dopa Oxidase Activity of Human Tyrosinase

**DOI:** 10.3390/ijms19082156

**Published:** 2018-07-24

**Authors:** Raffaella Micillo, Julia Sirés-Campos, José Carlos García-Borrón, Lucia Panzella, Alessandra Napolitano, Conchi Olivares

**Affiliations:** 1 Department of Chemical Sciences, University of Naples Federico II, Via Cintia 4, I-80126 Naples, Italy; raffaella.micillo@unina.it (R.M.); panzella@unina.it (L.P.); 2 Department of Biochemistry and Molecular Biology, School of Medicine, University of Murcia and Instituto Murciano de Investigación Biosanitaria (IMIB), 30120 El Palmar, Spain; juliasc@um.es (J.S.-C.); gborron@um.es (J.C.G.-B.)

**Keywords:** caffeic acid, dihydrolipoic acid, tyrosinase, inhibition mechanism, dopa oxidase, skin lightening, depigmenting agent

## Abstract

Caffeic acid derivatives represent promising lead compounds in the search for tyrosinase inhibitors to be used in the treatment of skin local hyperpigmentation associated to an overproduction or accumulation of melanin. We recently reported the marked inhibitory activity of a conjugate of caffeic acid with dihydrolipoic acid, 2-*S*-lipoylcaffeic acid (LCA), on the tyrosine hydroxylase (TH) and dopa oxidase (DO) activities of mushroom tyrosinase. In the present study, we evaluated a more lipophilic derivative, 2-*S*-lipoyl caffeic acid methyl ester (LCAME), as an inhibitor of tyrosinase from human melanoma cells. Preliminary analysis of the effects of LCAME on mushroom tyrosinase indicated more potent inhibitory effects on either enzyme activities (IC_50_ = 0.05 ± 0.01 μM for DO and 0.83 ± 0.09 μM for TH) compared with LCA and the reference compound kojic acid. The inhibition of DO of human tyrosinase was effective (Ki = 34.7 ± 1.1 μM) as well, while the action on TH was weaker. Lineweaver–Burk analyses indicated a competitive inhibitor mechanism. LCAME was not substrate of tyrosinase and proved nontoxic at concentrations up to 50 μM. No alteration of basal tyrosinase expression was observed after 24 h treatment of human melanoma cells with the inhibitor, but preliminary evidence suggested LCAME might impair the induction of tyrosinase expression in cells stimulated with α-melanocyte-stimulating hormone. All these data point to this compound as a valuable candidate for further trials toward its use as a skin depigmenting agent. They also highlight the differential effects of tyrosinase inhibitors on the human and mushroom enzymes.

## 1. Introduction

Melanin pigmentation is believed to be one of the main determinants of sensitivity to ultraviolet light and susceptibility to sun damage [1]. Under physiological conditions, its synthesis is restricted to melanosomes, membrane-bound organelles located in melanocytes, and is under complex regulatory control by multiple agents interacting via pathways activated by receptor-dependent and -independent mechanisms [2,3]. An overproduction or accumulation of melanin can lead to a local excess of pigmentation (or “hypermelanosis”) associated with disorders such as melasma, lentigo, or postinflammatory hyperpigmentation [2,4,5] whose medical and aesthetical impact has prompted a constant search for new nontoxic depigmenting agents [6,7,8,9,10].

Since control of pigmentation depends on several factors that can be regulated individually or concomitantly, different control points are possible, e.g., tyrosinase activity, expression, and stability, the use of chemicals to inhibit the reaction pathway leading to melanin, or interference with melanosome transfer [11,12,13]. One of the most common approaches involves the use of inhibitors of tyrosinase (EC 1.14.18.1). This enzyme catalyzes the initial steps of melanogenesis [13,14,15], namely the hydroxylation of the monophenol l-tyrosine to the *o*-diphenol l-3,4-dihydroxyphenylalanine (DOPA), and the oxidation of DOPA to the corresponding *o*-quinone, dopaquinone, via an electron exchange with copper atoms present in the active site [16,17,18]. These two tyrosinase activities are termed cresolase and catecholase activity or, more specifically in the case of melanogenesis, tyrosine hydroxylase (TH) and DOPA oxidase (DO) activity. Oxidative polymerization of products deriving from intramolecular cyclization of dopaquinone leads to eumelanin pigments [19,20].

A critical issue in the search for tyrosinase inhibitors is that most compounds, tested preliminarily on mushroom tyrosinase, proved to be noneffective on human tyrosinase [13,21,22,23]. Currently only a few tyrosinase inhibitors have been further developed for cosmetic and health care purposes as several factors such as cytotoxicity, solubility, cutaneous absorption, and stability should be considered as well.

**Figure 1:**
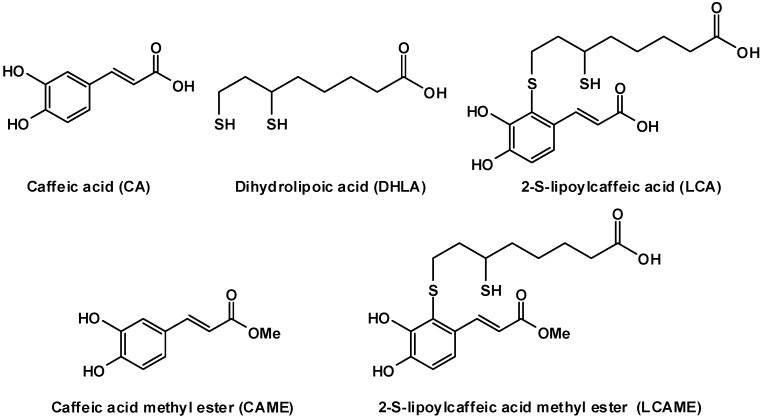
Structures of the compounds used in this study.

Bioinspired structural manipulations of catechol systems from natural sources have been pursued as a strategy to potentiate their properties for several applications in biomedicine and materials science [24]. In a previous paper [25], we reported the inhibitory activity of a conjugate of caffeic acid (CA) with dihydrolipoic acid (DHLA). Together with its oxidized form lipoic acid (LA), DHLA behaves as a powerful antioxidant [26,27] that can exert its functions at membrane level and in aqueous phases of cytoplasm, being soluble both in fats and water. The structure of these compounds is shown in Figure 1. Both DHLA and LA per se have been reported to block the expression of microphthalmia-associated transcription factor (Mitf) [28], a master regulator of the expression of melanogenic proteins [29]. DHLA was shown to affect melanogenesis by trapping dopaquinone and has therefore been included in topical formulations [30].

The *S*-conjugate of DHLA and caffeic acid, the 2-S-lipoylcaffeic acid (LCA), showed a half maximal inhibitory concentration (IC_50_) value of 3.22 and 2.0 μM for the DO and TH activity of the mushroom enzyme, respectively, being a promising lead structure for the development of new catechol-based bioinspired tyrosinase inhibitors [25]. Moreover, LCA was shown not to be a substrate of tyrosinase, a critical issue for the toxicity of depigmenting agents in vivo as fully appreciated further to the case of rhododendrol, a phenolic depigmenting agent whose oxidation products generated by the action of tyrosinase proved to be toxic and able to induce leukoderma [31,32,33,34,35]. The nature of tyrosinase inhibition was explored using Lineweaver–Burk plots suggesting a mixed type mechanism. Under the same conditions, caffeic acid did not show any effect, indicating that the inhibitory activity stems likely from the insertion of DHLA in the molecular scaffold.

Caffeic acid is a substrate of tyrosinase allegedly for its similarity to l-DOPA [36], while its derivatives containing amino acid residues such as the caffeoyl-prolyl-hydroxamic acid (CA-Pro-NHOH) proved to be good tyrosinase inhibitors [37], possibly because of the copper-chelating properties and the hydrophobicity that provides the proper structure for binding to the active site of tyrosinase. Actually, amino acids containing hydrophobic aliphatic side chains like leucine are known to play an important role in tyrosinase inhibition, and amino acids containing a hydrophobic aromatic ring like phenylalanine can interact with tyrosinase because of its similarity to tyrosine, the natural substrate of the enzyme [38]. Also amidic derivatives of caffeic acid with serine or lysine, in particular *N*-caffeoyl-*O*-acetylserine methyl ester, showed strong tyrosinase inhibitory activity [39].

On this basis, in the present study we examined the ability of LCA to inhibit tyrosinase from HBL human melanoma cells. In order to assess the contribution of the addition of hydrophobic groups to the tyrosinase inhibitory potency, the methyl ester of LCA, the 2-*S*-lipoylcaffeic acid methyl ester (LCAME), was prepared and the dose-activity profile was analyzed in comparison with the parent compound and a reference tyrosinase inhibitor as kojic acid. Moreover, Lineweaver–Burk plots were obtained to disclose the mechanism of inhibition. The effects on tyrosinase expression and cell viability were also analyzed.

## 2. Results and Discussion

### 2.1. Preparation of LCAME

LCAME was prepared by a procedure previously developed for LCA [25], involving in situ generation of the *o*-quinone of caffeic acid ester by the regioselective hydroxylation of *p*-coumaric acid methyl ester with 2-iodoxybenzoic acid (IBX), followed by addition of DHLA. LCAME was obtained in pure form in 52% yield after preparative High Pressure Liquid Chromatography (HPLC) purification and subjected to complete spectral characterization.

### 2.2. Inhibition of Mushroom Tyrosinase Activities by LCAME

The inhibition properties of LCAME were preliminarily investigated on mushroom tyrosinase by spectrophotometric monitoring of dopachrome formation, a method which is routinely used for evaluation of the activity of potential tyrosinase inhibitors [13,21,22]. l-DOPA was used as the substrate to test DO activity while l-tyrosine was used to test TH activity.

LCAME was incubated in 50 mM phosphate buffer in the presence of mushroom tyrosinase (20 U/mL) at room temperature. After 10 min, l-DOPA or l-tyrosine (1 mM final concentration) were added and the absorbance at 475 nm was measured at different times over 10 min. For comparative purposes the effects of CAME were evaluated as well.

Table 1 reports the IC_50_ values obtained for LCAME and CAME in comparison with those reported for LCA [25]. For the DO activity of tyrosinase, the inhibitory potency of LCAME was much higher than that of CAME or LCA. Concerning TH activity, LCAME was as well the most effective inhibitor, with LCA showing a comparable potency, whereas CAME was far less active. Comparing the effects of LCAME and LCA, it appears that the former is more active on DO activity than on TH activity whereas the reverse holds for LCA. These findings are in keeping with the occurrence of subtle differences in the structural requirements for efficient recognition of mono- and diphenols by tyrosinase, previously described for the mammalian enzyme [40]. Moreover, it should be cautioned that promising results with fungal enzyme are not expected to be straightforwardly confirmed using mammalian tyrosinase.

In order to investigate the effect of pre-incubation on the inhibitory activity, we performed a similar assay adding l-DOPA immediately after LCAME at a final concentration of 0.050 μM. Under these conditions a decrease of the inhibition from 50 ± 2% to 42 ± 1% (*p* = 0.02) was observed.

Separate experiments aimed at investigating the possibility that the decreased absorbance at 475 nm observed with the caffeic acid derivatives under study might actually be due to a redox exchange process or to addition to dopachrome. The inhibitor was therefore added to the reaction mixture 10 min after the start of the reaction of l-DOPA, when dopachrome formation was at its maximum, but no effect was observed on the intensity of the absorbance at 475 nm.

Some tyrosinase inhibitors are also substrates of the enzyme and this is a critical issue because their oxidation can lead to the formation of highly reactive, cytotoxic *o*-quinones [22,41,42]. To rule out this possibility, either LCAME or CAME at 100 μM were exposed to mushroom tyrosinase, in the absence of l-DOPA, and after 10 min, the reaction mixtures were analyzed by HPLC. In the case of LCAME, no significant consumption of the inhibitor was observed, suggesting that it is not a substrate of the enzyme. The same result was obtained in the presence of l-DOPA. In the case of CAME, the consumption of the inhibitor was almost complete but it was extremely low in the presence of l-DOPA, suggesting a competition between the two compounds as substrates.

### 2.3. Effect of Lipoylconjugates on Human Tyrosinase Activity

In further experiments, the effect of LCAME and LCA was investigated on extracts from HBL human melanoma cells expressing tyrosinase, and the inhibition of DO activity was evaluated. DO activity was determined according to Winder and Harris [43] with some minor modifications: cell extracts were incubated with 3-methyl-2-benzothiazolinone hydrazone (MBTH) and absorbance at 490 nm was measured every 10 min for 1 h.

**Figure 2:**
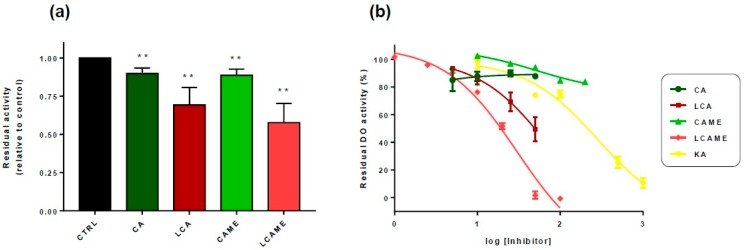
Effect of the caffeic acid derivatives included in this study on the DO activity of tyrosinase from HBL human melanoma cells. The oxidation of l-DOPA by cell-free HBL cell extracts was monitored spectrophotometrically at 490 nm for 1 h, in the presence of MBTH, as described in Materials and Methods. (**a**) Residual DO activity of HBL extracts incubated with 25 µM CA, LCA, CAME or LCAME. Data are represented as residual activity relative to the control activity measured in identical conditions but in the absence of the inhibitors. The results shown are the mean ± SEM of 4 independent experiments, ** *p* < 0.01. (**b**) Dose-response curves for DO activity inhibition. The DO activity of HBL cell extracts was measured in the presence of several concentrations (0 to 1000 µM) of the indicated compounds. Residual activities were determined as above and plotted against the logarithm of the inhibitor concentration (µM). Data are shown as the mean ± SEM of three independent experiments. KA = kojic acid.

These experiments showed a significant inhibitory effect on DO activity of both LCA and LCAME employed at a fixed concentration of 25 μM, whereas the corresponding compounds lacking the lipoic acid moiety showed a statistically significant but lower inhibition activity (Figure 2a). Next, the dose dependence profile for inhibition of DO activity was established by determining the residual DO activity at a fixed l-DOPA concentration, in the presence of increasing concentrations of the inhibitors. The results are shown in Figure 2b, expressed as percentage of the maximal activity (in the absence of the inhibitors). In the presence of LCA and LCAME, residual activity was far lower than that of kojic acid (KA), the reference DO inhibitor. IC_50_ values were evaluated as 76 μM for LCA, and as low as 30 μM for LCAME (Table 2).

On the other hand, incubation of LCAME at different concentrations in the presence of HBL cell lysates and MBTH did not induce any change in the absorbance at 490 nm for over 1 h, suggesting that the compound is not a substrate for human tyrosinase. Overall, these results indicated LCAME as the most promising inhibitor and further experiments mostly focused on this compound.

**Figure 3:**
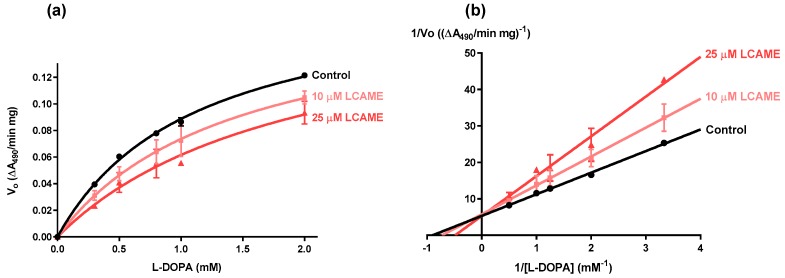
(**a**) Michaelis-Menten and (**b**) Lineweaver-Burk plots for human tyrosinase DO activity in the presence of LCAME. The DO activity of HBL cell-free extracts was assayed using concentrations of l-DOPA ranging from 0.1 to 2.0 mM as described in Materials and Methods, in the absence or presence of 10 or 25 µM of LCAME. The reaction rate, normalized for protein content, was represented vs. the substrate concentration (**a**), and the corresponding double reciprocal plots were obtained (**b**) using the GraphPad Software for Michaelis–Menten kinetics.

In order to determine the type of inhibition induced by LCAME, DO activity was measured at varying concentrations of l-DOPA (from 0.1 to 2.0 mM), in the presence of two fixed concentrations of LCAME (10 or 25 μM) (Figure 3a) and Lineweaver–Burk plots were obtained (Figure 3b). No significant changes in Vmax between the control conditions and the two series tested in the presence of the inhibitor were detected and a progressive increase in the apparent K_m_ (K_m_^app^) was observed (Table 3), strongly suggesting a competitive inhibition. Analysis of the data assuming Michaelis-Menten kinetics and a competitive inhibition profile where K_m_^app^ = K_m_ (1 + [I]/Ki), yielded a Ki of 34.7 ± 1.1 μM. On this basis it appears that introduction of a methyl group in LCA increased its inhibitory potency towards human tyrosinase.

### 2.4. Effects on HBL Cell Viability

**Figure 4:**
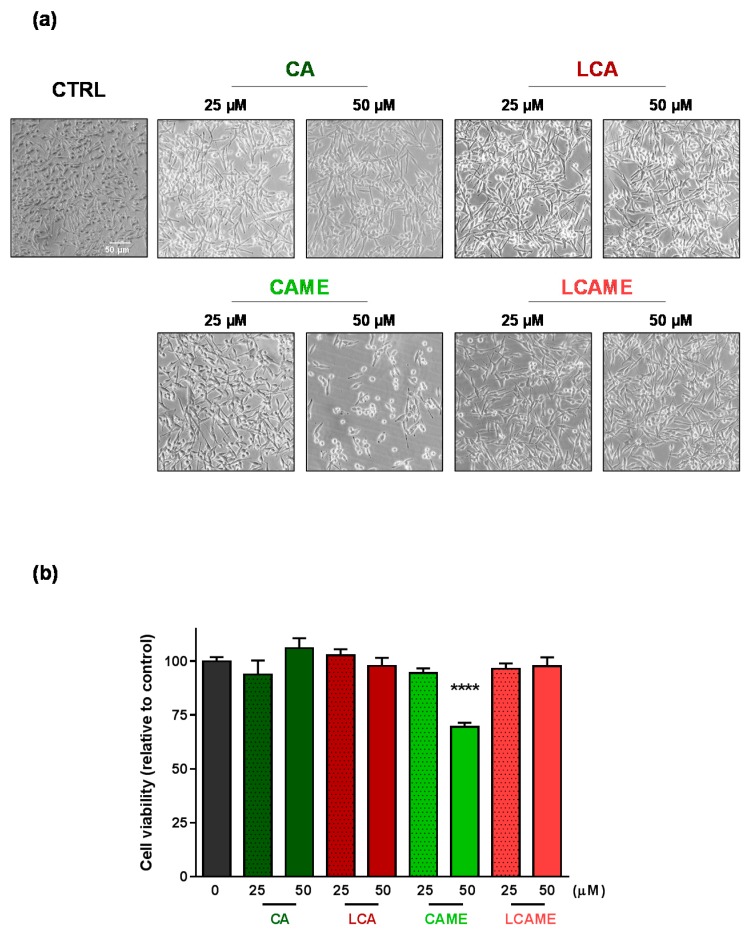
Effect of caffeic acid derivatives on the viability of HBL cells. Cells were incubated for 24 h with the inhibitors at 25 or 50 µM. (**a**) Phase contrast microscopy images. Representative images are shown (*n* = 8) Bar size: 50 μm. (**b**) MTT viability assay. Cells were grown in 96-well plates to ~80% confluence in the presence or absence of the indicated compounds. After 24 h, MTT was added (1 mg/mL final concentration) and maintained in the culture media for 4 h. Cells were PBS-washed twice and solubilized in DMSO. Absorbance at 562 nm was measured and represented as a percentage of the control value. The mean ± SEM of 8 independent experiments is shown. **** *p* < 0.0001.

The effect of the inhibitors on cell viability was evaluated by phase contrast microscopy and by means of the 3-(4,5-dimethyl-2-thiazolyl)-2,5-diphenyl-2*H*-tetrazolium bromide (MTT) assay. HBL cells were treated with the compounds (25 or 50 μM) for 24 h. At the end of the incubation period, phase contrast images were taken and the MTT assay was performed to test the cytotoxicity of each compound. Cells treated with CA, LCA, or LCAME exhibited a similar morphology than control, untreated cells (Figure 4a), whereas treatment with 50 μM CAME induced a significant change in the cells, which became rounded, indicative of detectable toxicity. Concerning their proliferation, data showed that CA, LCA, or LCAME were not cytotoxic at the concentrations used. Conversely, CAME reduced cell viability when employed at the highest concentration tested (50 μM) (Figure 4b) in keeping with the observed changes in phase contrast micrographs.

### 2.5. Effect of Inhibitors on Human Tyrosinase Expression

Both DHLA and LA have been reported to block the expression of Mitf [28], a master melanocyte transcription factor that upregulates the expression of genes encoding for tyrosinase and other melanogenic proteins [29]. Mitf expression is normally activated via cAMP signaling following binding of α melanocyte-stimulating hormone (αMSH) to the melanocortin 1 receptor (MC1R), thus accounting for the induction of tyrosinase activity in melanocytes stimulated by αMSH [44,45]. Accordingly, it was of interest to determine whether in addition to the competitive inhibition of tyrosinase activity observed in the kinetic analysis described above, LCAME might also lead to reduced expression of tyrosinase in cells cultured in the presence of the compound. To this end, we used HBL human melanoma cells as a convenient cellular model. HBL cells are wild type for the *MC1R*, *NRAS* and *BRAF* genes, which allows them to respond to αMSH by activating the cAMP and ERK pathways responsible for the regulation of MITF expression and stability, much like normal human melanocytes [46] Accordingly, they have been widely used to study the molecular mechanisms accounting for the physiological responses to αMSH [47,48,49]. Moreover, their high basal pigmentation and tyrosinase activity make them well-suited for the analysis of potential depigmenting agents.

**Figure 5:**
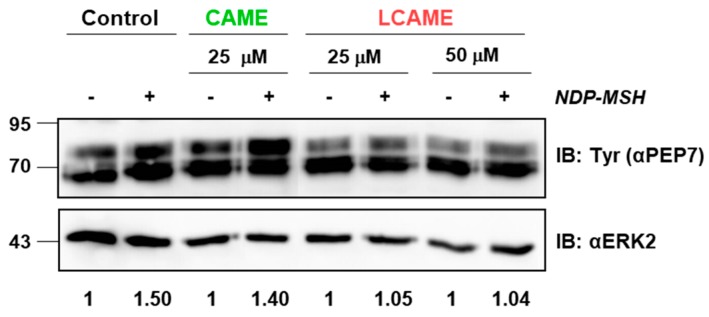
Effect on tyrosinase expression of CAME (25 µM) and LCAME (25 and 50 µM). HBL melanoma cells were incubated with or without the indicated compounds and stimulated with NDP-MSH (10^−7^ M). After 48 h of treatment, tyrosinase levels were compared by Western blotting, performed as described in Materials and Methods. Comparable loading was assessed by staining the membranes with an anti-ERK2 antibody. For each experimental condition, the fold-increase of tyrosinase abundance upon NDP-MSH treatment relative to the corresponding control was determined by densitometric analysis (ImageJ software) and is shown below the figure.

Control HBL cells or cells grown in the presence of CAME (25 µM) or LCAME (25 or 50 µM) were stimulated for 48 h with a potent analogue of αMSH ([Nle^4^,D-Phe^7^]-α-MSH (NDP-MSH), 10^−7^ M). Use of the 50 μM concentration of CAME was avoided since this concentration was somewhat cytotoxic as shown above. Expression of tyrosinase was then evaluated by Western blot using a specific antibody directed against tyrosinase (αPEP7). Tyrosinase displayed the expected electrophoretic pattern corresponding to the presence of several *N*-glycosylation forms [50]. No significant changes in basal tyrosinase expression were observed for cells treated with either CAME or LCAME (Figure 5). Moreover, in control cells and in cells grown in the presence of 25 µM CAME, a compound lacking a lipoic acid-derived moiety, tyrosinase expression was similarly induced by NDP-MSH. Conversely, LCAME apparently blocked NDP-MSH-mediated stimulation of tyrosinase, suggesting that its lipoic acid moiety retained the ability to interfere with Mitf expression. However, other mechanisms such as interference with activation and/or functional coupling of the melanocortin 1 receptor responsible for αMSH actions cannot be ruled out and a complete study of these possibilities is beyond the scope of this work. Accordingly, interference of LCAME with MITF expression or activity remains speculative at this stage. In any case, this preliminary observation deserves confirmation using cultured normal human melanocytes and further mechanistic analysis, as it may further support the potential use of LCAME as a skin depigmenting agent. Indeed, LCAME may act at two different levels, on one hand by inhibiting preexisting tyrosinase by a competitive mechanism, and on the other hand by interfering with αMSH-dependent induction of tyrosinase expression.

## 3. Materials and Methods

### 3.1. Materials

Iodobenzoic acid, oxone^®^, (±)-lipoic acid (LA), sodium borohydride, *p*-coumaric acid, l-tyrosine, l-3,4-dihydroxyphenylalanine (l-DOPA)**,** bovine serum albumin, phenylmethylsulfonylfluoride (PMSF), Igepal CA-630 NP 40, and 3-methyl-2-benzothiazolinone hydrazone hydrochloride hydrate (MBTH) were purchased from Sigma-Aldrich (Milan, Italy). Trichloroacetic acid, glycine, Tris, EDTA, and monosodium and disodium phosphate were from Merck (Darmstadt, Germany). Acrylamide, bisacrylamide, TEMED, Tween 20, and ammonium persulfate were obtained from Bio-Rad (Hercules, CA, USA).

High-performance liquid chromatography (HPLC) grade solvents (VWR, Milan, Italy) and Milli-Q^®^ water were used. 

2-Iodoxybenzoic acid (IBX) [51], DHLA [52], 2-*S*-lipoylcaffeic acid (LCA) [25] were synthesized as reported.

### 3.2. Methods

Nuclear magnetic resonance (NMR) spectra were recorded at 400 MHz in deuterated solvents on a Bruker instrument (Milan, Italy).

Assays of mushroom tyrosinase activity were performed recording spectra on a UV Jasco V-730 Spectrophotometer (Lecco, Italy).

HPLC purification was performed on an Agilent 1100 binary pump instrument (Agilent Technologies, Milan, Italy) equipped with a UV detector set at 254 nm using an Econosil C18 column (22 mm × 250 mm, 10 μm) and 0.1% formic acid: methanol 35:65 *v*/*v* as the eluant, at 15 mL/min.

HPLC analyses for determination of the consumption of CAME and LCAME in the presence of mushroom tyrosinase were performed on a Agilent 1100 binary pump instrument equipped with a UV-visible detector using an octadecylsilane-coated column, 250 mm × 4.6 mm, 5 μm particle size (Phenomenex Sphereclone ODS, Bologna, Italy) at 0.7 mL/min, using the following gradient: 0.1% formic acid (eluant a)/ methanol (eluant b): 40% b, 0–10 min; from 40 to 80% b, 47.5–52.5 min; from 80 to 40% b, 52.5–57.5 min. Detection wavelength was set at 280 nm.

Liquid chromatography-mass spectrometry (LC-MS) analysis of LCAME was performed on an Agilent HPLC 1100 VL instrument (Agilent Technologies, Milan, Italy), equipped with an electrospray ionization source (positive ion mode, ESI+). An Eclipse XBD-C18 column (150 mm × 4.60 mm, 5 μm) was used, adopting the same eluant used for the HPLC analysis (flow rate: 0.4 mL/min). Conditions were set up as following: nebulizer pressure 50 psi; drying gas (nitrogen) flow 10 L/min, 350 °C, and capillary voltage 4000 V.

### 3.3. Synthesis of p-Coumaric Acid Methyl Ester

*p*-coumaric acid methyl ester was prepared by reacting *p*-coumaric acid (600 mg) in methanol (6 mL) with 96% sulfuric acid (600 μL) under reflux. After 2 h the mixture was diluted with water, extracted with ethyl acetate, and washed twice with a 5% sodium bicarbonate solution. The organic layer was dried over sodium sulfate and taken to dryness to afford the product (410 mg, 58% yield) as a pale-yellow powder.

### 3.4. Synthesis of 2-S-Lipoylcaffeic Acid Methyl Ester (LCAME)

A solution of *p*-coumaric acid methyl ester (180 mg, 0.90 mmol) in methanol (12 mL) was treated with IBX (384 mg, 1.38 mmol) under vigorous stirring at room temperature. After 7 min a solution of DHLA (774 mg, 3.66 mmol) in methanol (12 mL) was added dropwise, and after additional 15 min the reaction mixture was concentrated and purified by preparative HPLC (209 mg, 52% yield).

ESI+/MS: *m*/*z* 401 ([M + H]^+^); UV: λ_max_ (CH_3_OH) 251, 318 nm; ^1^H-NMR (CD_3_OD):δ (ppm) 1.38 (m, 1H), 1.54 (m, 1H), 1.54 (m, 2H), 1.42 (m, 1H), 1.62 (m, 1H), 1.78 (m, 1H), 1.81 (m, 1H), 2.26 (m, 2H), 2.88 (m, 1H), 2.90 (m, 1H), 2.96 (m, 1H), 3.78 (s, 3H), 6.33 (d, J = 16 Hz, 1H), 6.84 (d, J = 8.4 Hz, 1H), 7.22 (d, J = 8.4 Hz, 1H), 8.40 (d, J = 16 Hz, 1H). ^13^C NMR (CD_3_OD): δ (ppm) 25.7 (CH_2_), 27.6 (CH_2_), 34.1 (CH_2_), 34.7 (CH_2_), 39.3 (CH_2_), 39.6 (CH_2_), 41.3 (CH), 57.5 (CH_3_), 117.0 (CH), 117.1 (CH), 120.0 (CH), 122.4 (C), 130.7 (C), 145.3 (CH), 148.3 (C), 148.6 (C), 169.6 (C), 177.4 (C).

### 3.5. Mushroom Tyrosinase Activity Inhibition Assay

Stock methanolic solutions of LCAME or CAME, ranging in concentration from 0.02 to 20 mM concentration were prepared dissolving a proper amount of the compound of interest in methanol. 100 μL of these solutions were added to 2 mL of 50 mM phosphate buffer at pH 6.8 (final concentrations range: 0.001–1 mM), in the presence or in the absence of 20 U/mL mushroom tyrosinase. After 10 min incubation at room temperature, 20 μL of a 100 mM solution of l-DOPA or l-tyrosine in 0.6 M HCl, were added (final concentration: 1 mM). Spectrophotometric analysis was performed by measuring the absorbance at 475 nm for 10 min at 2 min intervals. In control experiments, the reaction was run in the absence of LCAME. When required, the experiment was performed as above but adding l-DOP A soon after the addition of LCAME (0.050 μM). In separate experiments, the assay was run as above with LCAME or CAME at 100 μM, in the presence or absence of l-DOPA, and after 10 min the mixture was analyzed by HPLC.

### 3.6. Cell Culture

HBL cells were kindly provided by Prof. G. Ghanem (Laboratory of Oncology and Experimental Surgery, Université Libre de Bruxelles, Brussels, Belgium). Cells were grown in a water-saturated 5% CO_2_ atmosphere in plates of the required size to ~80% confluence. The culture medium was DMEM-GlutaMAX^TM^ (Gaithersburg, MD, USA) supplemented with 10% fetal bovine serum, penicillin (100 U/mL) and streptomycin (100 μg/mL).

### 3.7. Enzyme Activity Determination

Cells were solubilized in 1% Igepal, 1% phenylmethylsulfonyl fluoride, 50 mM phosphate buffer (pH 6.8) and centrifuged at 2000 rpm for 5 min.

DO activity determinations were performed according to Winder and Harris [43] with minor modifications. Briefly, cell extracts (usually 50–100 µg protein/assay) were incubated with 5 mM MBTH and 2.5 mM L-DOPA in 50 mM phosphate buffer pH 6.8 in the presence of the indicated concentrations of the compound of choice in a 96-wells plate. DO activity was measured spectrophotometrically in a microplate reader every ten minutes for one hour and was expressed as the slopes calculated with the GraphPad Prism 7 software (Linear Regression, San Diego, CA, USA).

For the analysis of kinetic parameters, the assays were run as above, using different concentrations of l-DOPA (from 0.1 to 2.0 mM) and of the inhibitor (0, 10 and 25 μM) to build the Lineweaver–Burk plot.

### 3.8. Immunochemical Techniques

After protein separation by SDS-PAGE, they were transferred from the gel to the membrane and immunochemical detection with anti-PEP7 was performed as previously described [53]. Briefly, the filters were blocked for 1 h in 2% bovine serum albumin in 1% Tween 20 PBS and incubated overnight at 4 °C with primary antibodies (1:4000 dilution). The filters were washed and further incubated with peroxidase-labeled mouse anti-rabbit IgG (1:5000 dilution). Staining and detection were done with ECL Plus Western Blotting Detection System chemiluminescent substrate (Thermo Fisher Scientific, Waltham, MA, USA). Images were acquired in a Fusion Solo.6S system (Vilber-Lourmat, Marne La Vallée, France). Signal intensities were estimated with ImageJ (National Institutes of Health, Bethesda, MA, USA) in independent blots, and normalized with the corresponding loading controls (obtained by staining the membranes with an anti-ERK2 antibody).

### 3.9. MTT Assay

Cell viability was measured by the MTT assay [54]. HBL cells were seeded into the wells of a 96-wells plate at a density of 10,000 cells in 200 μL cell culture media. The seeded cells were incubated for 18 h at 37 °C, then treated with the compounds (25 or 50 μM) for 24 h or left untreated. At the end of the incubation period, images were taken and the MTT assay was performed to test the cytotoxicity of the different caffeic acid derivatives.

### 3.10. Statistical Analysis

Results are given as mean ± SEM for experiments performed at least twice, with independent duplicates or triplicates (*n* ≥ 4), unless specified otherwise. Statistical significance was assessed with an unpaired two-tailed Student’s *t*-test, using the GraphPad Prism package (GraphPad Software, San Diego, CA, USA). *p* values of <0.05 were considered statistically significant.

## 4. Conclusions

2-*S*-lipoylcaffeic acid (LCA), the *S*-conjugation product of caffeic acid and dihydrolipoic acid, and its methyl ester derivative (LCAME) inhibited the TH and DO activities of mushroom tyrosinase, with IC_50_ values much lower than those obtained for the reference inhibitor kojic acid. Under the same conditions, caffeic acid (CA) and caffeic acid methyl ester (CAME) did not show any effect, pointing to a critical role of the lipoyl moiety in determining the inhibitory activity.

The higher inhibitory potency of LCAME compared with LCA further points to improvement of the inhibitory action as a result of enhancement of lipophilicity.

The inhibitory activity of lipoyl adducts was maintained on the DO activity of human tyrosinase, with LCAME being more potent than LCA, whereas the effects of both compounds on the TH activity were much smaller.

LCAME is not substrate of human or mushroom tyrosinase. In addition, the lack of toxicity of LCAME on HBL human melanocytic cells further supports the perspective of its exploitation for control of hyperpigmentation.

Analysis of the mechanism of action of LCAME on human tyrosinase indicated a competitive mechanism. However, it is also possible that the lipoyl moiety might impair Mitf induction in αMSH-stimulated cells, thus contributing to tyrosinase inhibition by an additional mechanism based on decreased tyrosinase gene expression.

The results of this study point to lipoyl conjugates, in particular LCAME, as good candidates for further trials toward their use as skin depigmenting agent.

## Figures and Tables

**Table 1:** IC_50_ values for caffeic acid methyl ester (CAME) and lipoyl caffeic acid methyl ester (LCAME) for Dopa Oxidase (DO) and Tyrosine Hydroxylase (TH) activities of mushroom tyrosinase.

Compound	IC_50_ (DO Activity)	IC_50_ (TH Activity)
**CAME**	155 ± 7 μM	79 ± 7 μM
**LCAME**	0.05 + 0.01 μM	0.83 ± 0.09 μM
**LCA** [25]	3.22 ± 0.02 μM	2.0 ± 0.1 μM

**Table 2:** IC_50_ values for selected compounds versus DO activity of human tyrosinase.

Compound	IC_50_ (Mean ± SEM, μM)
**CA**	n.d.
**LCA**	76.2 ± 6.0
**CAME**	n.d.
**LCAME**	30.1 ± 1.5
**Kojic acid**	282.2 ± 1.8

IC_50_ values were calculated by a nonlinear regression of the semi-logarithmic plot (log[inhibitor] vs. response curves) of the data shown in Figure 2b using the GraphPad Prism software. Results are expressed as mean ± SEM of two independent assays. “n.d.” stands for “not determined”, as the inhibitory action of CAME was too low to enable an accurate estimate of the IC_50_, and CA did not significantly inhibit the DO activity of human tyrosinase at the concentrations tested.

**Table 3:** Effect of LCAME on the kinetic parameters for the DO activity of human tyrosinase.

[LCAME] (μM)	Vmax (∆A490/min mg)	Km (mM)
**0**	0.19 ± 0.01	1.12 ± 0.06
**10**	0.18 ± 0.02	1.43 ± 0.30
**25**	0.19 ± 0.03	1.95 ± 0.50

Results are expressed as mean ± SEM and were obtained by nonlinear regression of the data shown in Figure 3a using the GraphPad Prism software for Michaelis–Menten kinetics.

## Abbreviations

α-MSH	Melanocyte Stimulating Hormone
CA	Caffeic acid
CAME	Caffeic acid methylester
LCA	2-*S*-lipoylcaffeic acid
TH	Tyrosine hydroxylase
DHLA	Dihydrolipoic acid
DO	Dopa oxidase
DOPA	l-3,4-Dihydroxyohenylalanine
IBX	2-iodoxybenzoic acid
IC_50_	Half maximum inhibitory concentration
KA	Kojic acid
LA	Lipoic acid
LCAME	2-*S*-lipoylcaffeic acid methylester
MBTH	3-methyl-2-benzothiazolinone hydrazone
Mitf	Microphthalmia-associated transcription
MTT	3-(4,5-dimethyl-2-thiazolyl)-2,5-diphenyl-2*H*-tetrazolium bromide
PMSF	Phenylmethylsulfonylfluorid
